# Observations on an Extraordinary Case of Ruptured Uterus

**Published:** 1785

**Authors:** 


VII. *Obfervations on an extraordinary Cafe of ruptured Uterus. By* Andrew Douglas, *M. D. Member of the College of Phyficians, London; Phyfician and Man-midwife to the Lying-in Charity, inftituted* 1757, *and one of*

 *the*

*the Physicians to the Asylum.* 8vo. Johnson, *London*, 1785. 74 pages.

THE patient, whose case is the principal subject of this work, was a poor woman in her fourth pregnancy, thirty years of age, low in stature, and of a delicate constitution, but, in general, healthy. She was seized with slight labour pains on the 11th of September, 1784, and the day following, when our author was desired to visit her, he found her extremely restless, and complaining of excessive pain in the region of the pubis. The membranes had been ruptured eight hours, and the pains had continued to return regularly from that time; but the head of the child, though plainly to be felt, was not engaged within the brim of the pelvis. Each return of pain occasioned her to writhe and twist, as if suffering from violent cholic, in a manner very different from the urging throes of common labour: her pulse, however, was calm and regular; nor did she make any particular complaint, excepting of the pain about the pubis. This being the state of the case, our author saw no reason that could induce him to precipitate the delivery. He, therefore, contented himself with advising the patient patient to be kept cool, and giving ſuch other directions to the midwife as the caſe ſeemed to require.

About nine o'clock the ſame evening, the midwife informed him that the patient had had a diſcharge of blood from the vagina, which, however, did not continue; that the pains had been more ſevere for an hour or two, and apparently with ſome effect, but had ceaſed about half an hour after ſix o'clock, after which, retching, and an inceſſant craving for drink had come on.

Our author now found her with a pale and ghaſtly countenance. Her face was lengthened and bedewed with cold ſweat. Her pulſe was ſcarcely to be felt; and her breathing was ſhort and quick, with great anxiety: but ſtill ſhe complained of no particular pain, except about the pubis.

By examination in the common way he could diſtinguiſh nothing, excepting a round moveable ſubſtance, which he ſuppoſed to be the head of the child, but on introducing his hand, this ſubſtance fled before the tips of his fingers, and by following it, he, at laſt, found his hand in a cavity, which, in no ſort, reſembled that of the uterus. "I was then," ſays he, "forcibly

"and

"and painfully ftruck with the nature of the
"cafe; and on examining all round, with
"caution and gentlenefs, I could, with cer-
"tainty, determine that my hand was in the
"cavity of the abdomen: the child lying on
"the fore part; on the back part, the contracted
"uterus like an oblong ball; and the intef-
"tines hanging among my fingers. It is eafy
"to imagine how miferable I felt at this mo-
"ment. No hope of doing good to the
"woman, from the ufual event of fuch cafes;
"no poffibility of confulting with any of my
"brethren; and a neceffity of determining in-
"ftantly how I was to proceed."

As his hand was already in contact with the child, our author decided in favour of immediate delivery, which he effected without any confiderable difficulty. While his hand was in fearch of the feet of the child, he difcovered that the placenta was likewife in the abdominal cavity, where it had fo clung to the inteftines, that he was under the neceffity of introducing his hand a fecond time to detach it. This afforded him an opportunity of being ftill better inftructed in the nature of the injury. The uterus feemed to have been ruptured tranf-verfely on the lower and fore part, at fome diftance diſtance above where the vagina is connected with it: and it was more contracted than he thought poſſible, in the few hours which had elapſed ſince the accident. The hæmorrhage was not greater than is uſual in a common labour; but the patient ſtill felt the ſame ſevere and unremitting pain, as before delivery, at the lower part of her belly. With a view to alleviate this pain, an opiate was adminiſtered; her room was directed to be kept cool and airy, and her drink to be mint tea or gruel.—The next day (Sept. 13) ſhe was quite ſenſible; her nauſea and retchings were leſs troubleſome, and her complaint was no longer of her belly, but of her breaſt, pointing to the ſeat of the heart. Her pulſe was quick and ſmall, but very regular. In the evening her retchings returned to a violent degree, with great heat and reſtleſſneſs, but theſe ſymptoms terminated towards morning (Sept. 14) in a profuſe ſweat, and ſhe ſlept four hours.

Sept. 15th, her pulſe beat about one hundred ſtrokes in a minute with conſiderable firmneſs; in the evening ſhe had two ſtools, and complained of griping. Her urine had been voided freely and regularly from the day of her delivery.

—— 17th

—— 17th, a diarrhœa had come on in the night, with a violent pain from the pit of the ſtomach along the whole inteſtinal canal. She likewiſe complained of a pain and ſoreneſs extending acroſs from Ilium to Ilium. Her belly was tumid, and rather hard; and her pulſe was quick (one hundred and ten) with a degree of fulneſs. She had ſome thirſt and a tendency to delirium. She was directed to loſe eight ounces of blood, and to take a cathartic ſolution in mint water, with a few drops of laudanum.

—— 18th, the delirium was gone off, and the abdominal pain had abated ſoon after the bleeding. She had only one ſtool in the night, yet her pulſe was quicker (one hundred and twenty) than it had ever been.—The uſe of the ſolution was continued.

—— 19th, ſhe had purged only twice in the laſt twenty-four hours. She had ſlept ſome hours in the night; her tongue was moiſt, and her pulſe (one hundred and ſixteen) was ſtronger and firmer than the day before.

—— 20th, having had a return of the pain and purging in the night, an opiate clyſter was injected in the morning; after which ſhe had no return of either. Her pulſe was about ninetyninety-ſix.—On the 24th, ſhe was well enough to be taken out of bed, and on the 29th, her pulſe was at ſeventy-two, firm and regular.—Theſe particulars we have abridged from our author's accurate journal of the caſe, which is continued to the 27th of October, when the patient was able to walk to his houſe, and was perfectly recovered. On examination, at that time, there was nothing in the touch ſo different from what is obſerved in the natural ſtate, as could have occaſioned any idea of previous diſeaſe, had the complaint, with which ſhe had been afflicted, not been known.

This patient took no other nouriſhment during the firſt week, than whey, milk, and plain gruel. In the next period of ten days, her diet was only mended by a raw egg beat up with water and a little ſugar. In the third week, ſhe began to take broth and light pudding; and afterwards came, by degrees, to a fuller diet.

This hiſtory is followed by abſtracts of ſimilar caſes, collected from books, or communicated to the author by his friends.

Theſe caſes give riſe to a great deal of judicious reaſoning on the ſubject, for which we muſt refer our readers to the work itſelf, contenting ourſelves with quoting from it the following concluſions, which, our author thinks, are deducible from the facts he has related, viz.

"First, That in a rupture of the gravid "uterus, which has allowed a fœtus to "paſs into the cavity of the abdomen, the "caſe is not to be conſidered as abſolutely "hopeleſs.

"Secondly, That the danger of ſuch a caſe, "is as much in conſequence of the injury "which the viſcera ſuſtain from the child "remaining in the cavity of the abdomen, "as from that which is done to the uterus "itſelf.

"Thirdly, That the danger will generally "be in proportion to the time the child is "ſuffered to remain among the viſcera, and "to the ſuſceptibility of irritation which pre-"vails in the conſtitution of the patient at the "time.

"Fourthly, That delivery affords the only "proſpect of recovery to the patient, and "ſhould, therefore, be effected as ſoon as the "circumſtances will permit."

Although theſe circumſtances ſeem to be fairly deducible from what is advanced by our author, yet we find him candidly acknowledging, ledging, that in cafes of this fort, circumftances will frequently arife, which will baffle all reafoning from general principles. It is a matter of fome comfort, however, that amidft fuch accumulated danger, there ftill remains a probability of the patient's recovery: and as that feems, in a great meafure, to depend on the fpeedy removal of the child from among the vifcera, it is a point of the higheft importance, to be able early and certainly to determine, that the accident has really happened. And accordingly Dr. Douglas has endeavoured to bring into our view all the circumftances he has been able to collect, which are likely to affift in directing our judgement on this occafion.

